# Establishment of human corneal epithelial organoids for ex vivo modelling dry eye disease

**DOI:** 10.1111/cpr.13704

**Published:** 2024-07-03

**Authors:** Xichen Wan, Jiayu Gu, Xujiao Zhou, Qihua Le, Jingyuan Wang, ChangChang Xin, Zhi Chen, Yao He, Jiaxu Hong

**Affiliations:** ^1^ Department of Ophthalmology, Eye & ENT Hospital, State Key Laboratory of Molecular Engineering of Polymers Fudan University Shanghai China; ^2^ NHC Key laboratory of Myopia and Related Eye Diseases Shanghai China; ^3^ Shanghai Engineering Research Center of Synthetic Immunology Shanghai China; ^4^ Macao Institute of Materials Science and Engineering Macau University of Science and Technology Taipa Macau SAR China; ^5^ Department of Ophthalmology Children's Hospital of Fudan University, National Pediatric Medical Center of China Shanghai China

## Abstract

Dry eye disease (DED) is a growing public health concern affecting millions of people worldwide and causing ocular discomfort and visual disturbance. Developing its therapeutic drugs based on animal models suffer from interspecies differences and poor prediction of human trials. Here, we established long‐term 3D human corneal epithelial organoids, which recapitulated the cell lineages and gene expression signature of the human corneal epithelium. Organoids can be regulated to differentiate ex vivo, but the addition of FGF10 inhibits this process. In the hyperosmolar‐induced DED organoid model, the release of inflammatory factors increased, resulting in damage to the stemness of stem cells and a decrease in functional mucin 1 protein. Furthermore, we found that the organoids could mimic clinical drug treatment responses, suggesting that corneal epithelial organoids are promising candidates for establishing a drug testing platform ex vivo. In summary, we established a functional, long‐term 3D human epithelial organoid that may serve as an ex vivo model for studying the functional regulation and disease modelling.

## Graphical abstract

The functional, long‐term 3D human epithelial organoids were established from human corneal epithelium tissue, which recapitulated the cell lineages and gene expression signatures of corneal epithelium. The organoid can be induced to differentiated, and the ex vivo DED model was produced under hyperosmotic stress, providing a potential to explore the stem cell identity and keratinocyte function in DED ex vivo. Based on DED organoid model, the drug testing was performed, and organoids could precisely mimic drug treatment responses of corneal epithelium.

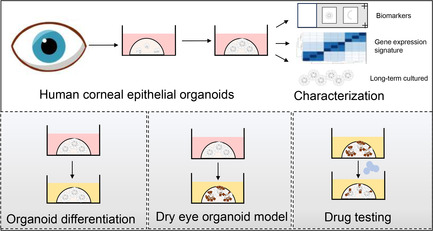

## 1 INTRODUCTION

The corneal epithelium, a stratified squamous non‐keratinized epithelium at the outermost layer of the cornea, is essential for homeostasis of the ocular surface. A healthy corneal epithelium prevents the damage of microbes and toxins, anchors the tear film, and provides a transparent medium for light to enter the eye.^1^ During homeostasis and injury, self‐renewal, proliferation, differentiation and centripetal migration of limbal epithelial stem cells repopulate the corneal epithelial cells.^2^

Dry eye disease (DED) is a common disorder that affects millions of people worldwide^3^, ^4^; therefore, numerous mechanistic studies and preclinical trials have been performed for disease treatment. However, studies of these diseases have mainly focused on conventional epithelial cell lines and animal models.^5^ Nevertheless, immortalized cell lines with specific cell types cannot model the complex 3D structure of the corneal epithelium; thus, it is challenging to investigate homeostasis and dysfunction of the human corneal epithelium in vitro.^5^, ^6^ In addition, the animal model is limited by anatomical and physiological differences from human eyes. Various efforts have been undertaken to develop alternative methods to closely mimic the human corneal epithelium ex vivo.^1^, ^7^

Organoids with three‐dimensional (3D) structures contain multiple cell types that can accurately mimic real tissues ex vivo and be used to track organ development and disease progression.^8^ Thus, the generation of human corneal epithelial organoids has the potential to enhance our understanding of corneal epithelium biology, model healthy and diseased epithelium from patients, and facilitate drug development and regenerative medicine.^9^ Although human corneal organoids have been established successfully from induced pluripotent stem cells,^10^, ^11^ adult stem cells (ASCs)‐derived corneal epithelial organoids also represent promising applications. In 2020, Higa et al. first described the generation of human corneal limbal organoids and demonstrated that these organoids could be engrafted to provide epithelial cells in a rabbit model of limbal deficiency.^12^ However, these organoids were cultured for 1 month in a serum‐containing medium. Serum promotes organoid senescence, which is not conducive for organoid growth, regulation, or long‐term passage. Thus, in our study, we aimed to achieve an ASC‐derived long‐term 3D human corneal epithelial organoid, which recapitulated the characteristics of the human corneal epithelium and provided a useful tool to model DED ex vivo.

## 2 RESULTS

### 2.1 Establishment and characterization of long‐term 3D human corneal epithelial organoids

**FIGURE 1:**
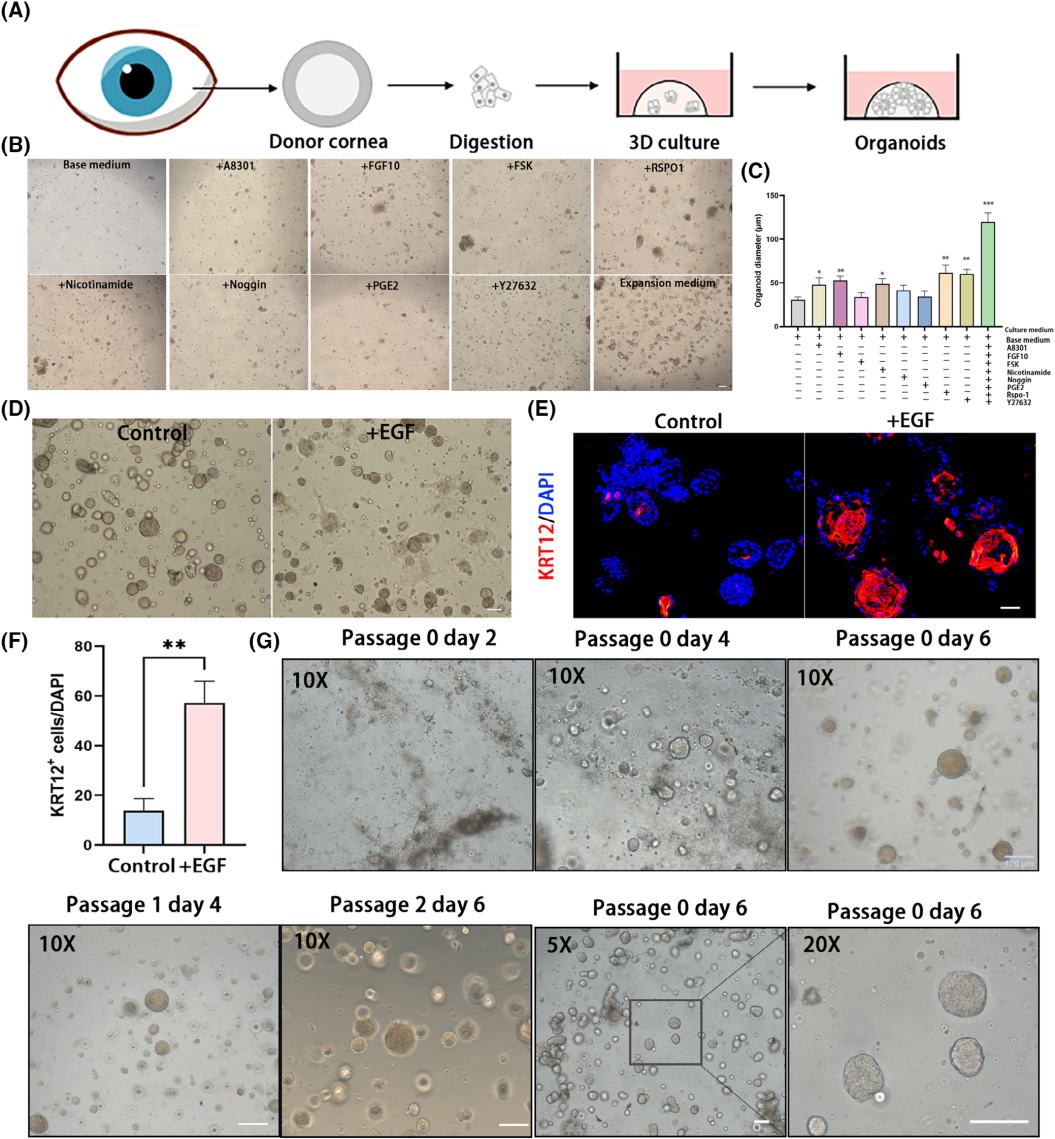
Establishment of the human corneal epithelial organoid from human cornea tissues. (A) Schematic representation of corneal epithelial organoid derivation from human cornea tissue. (B) Representative images of organoid outgrowth in 10 different culture mediums. Scar bar: 100 μm. (C) Quantitative analysis of organoid diameters in 10 different culture mediums. Data points represent biological replicates. Error bars indicate mean ± SD. *p < 0.05; **p < 0.01; ***p < 0.001. (D) Representative bright‐light images of organoid morphology in culture medium with or without EGF. Scar bar: 100 μm. (E) Immunofluorescent staining for organoid cultured in medium with or without EGF. Scar bar: 50 μm. (F) Quantitative analysis of the KRT12^+^ cells in medium with or with EGF. (G) Representative bright‐light images of organoid in expansion medium at passage 0–2. Scar bar: 100 μm.

The entire research objectives are showed in Figure S1. We optimized published conjunctival organoid and lacrimal gland organoid protocols to establish human corneal epithelial organoids from donor corneal tissues.^9^, ^13^ The human corneas were collected. After dissecting the sclera, the epithelium facing the plate and collagenase I were used for digestion. The epithelium was scraped and the cells were embedded in Matrigel for culturing (Figure 1A). Culture medium containing HEPES, Glutamax, N‐acetylcysteine, and B27 supplement in Advanced DMEM/F12 formed the base medium, and different growth factors were added in base medium to reveal the effect of single factors (Figure 1B). Supplementation of the transforming growth factor inhibitor A83‐01 (*p* = 0.027), fibroblast growth factor 10 (FGF10, *p* = 0.003), R‐spondin 1 (Rspo‐1, *p* = 0.005), nicotinamide (*p* = 0.011), the Rho‐associated kinase (ROCK) inhibitor Y‐27632 (*p* = 0.001) significantly accelerated organoid outgrowth (Figure 1C). Although organoid sizes slightly increased after treatment with the cyclic AMP/Protein kinase A activators forskolin (FSK), prostaglandin E2 (PGE2), and the bone morphogenetic protein (BMP) antagonist Noggin (Figure 1C), these signalling pathways are considered key factors in regulating corneal epithelium functions.^14^, ^15^ Thus, FSK, PGE2, and Noggin were also added to the culture medium, and this cocktail showed an obvious capacity to boost organoid expansion (*p* < 0.001; Figure 1C). However, upon addition of epidermal growth factor (EGF), the organoids tended to morph into flat shapes (Figure 1D). Immunofluorescence (IF) staining revealed that the expression of the differential corneal epithelial marker, keratin‐12 (KRT12), increased significantly in the medium with EGF (*p* = 0.002, Figure 1E,F). Therefore, we removed EGF and established an expansion medium (EM) to maintain the outgrowth of human corneal epithelial organoids. Utilizing EM, the seeded corneal epithelial cells formed spheroid structures within 2‐days of incubation (Figure 1G), and the organoids expanded to a diameter of ~150 μm within 6 days (Figure 1G).

**FIGURE 2:**
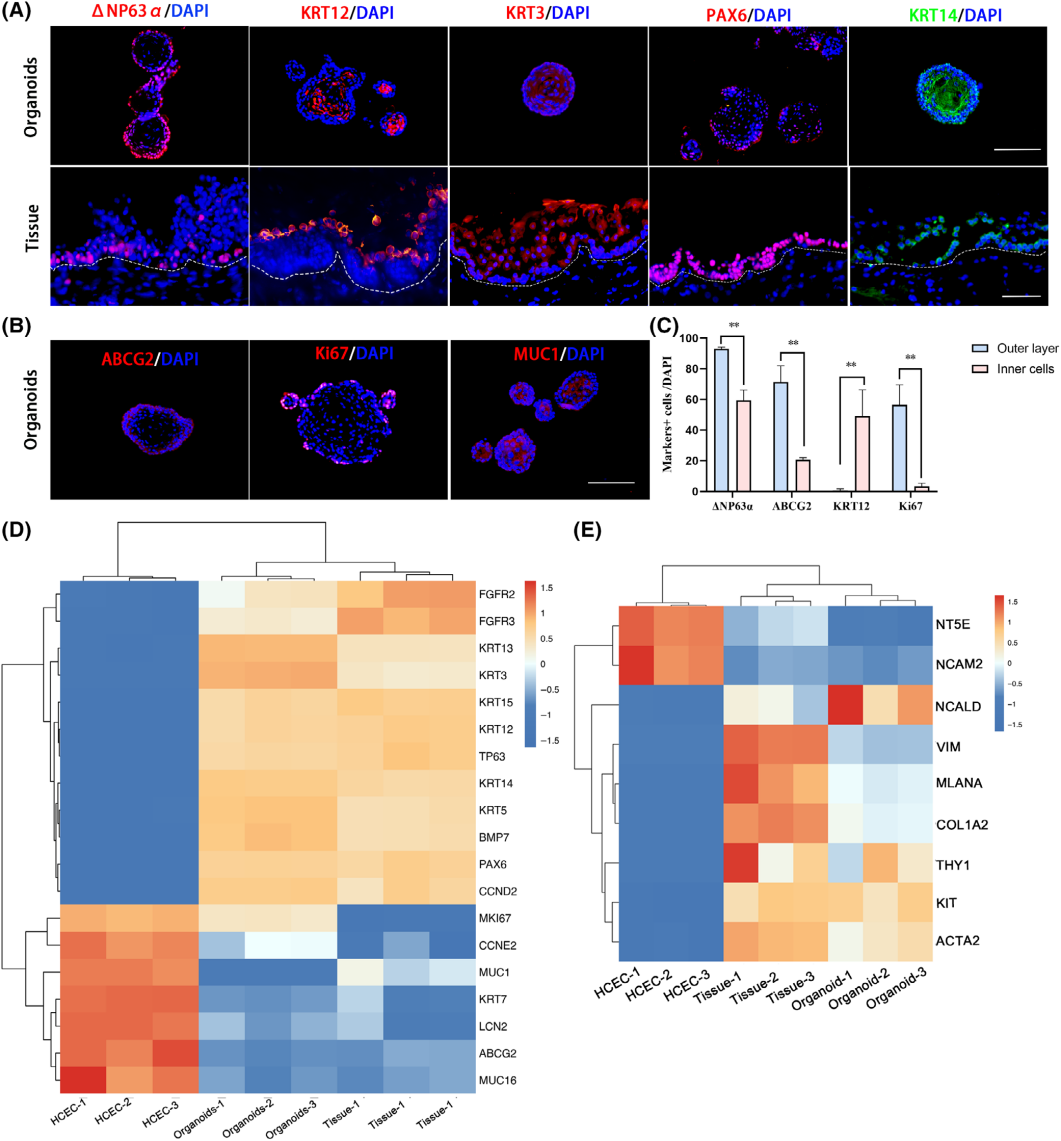
Mapping the biomarkers and gene expression pattern in human epithelial organoids and tissues. (A) Immunofluorescent staining for ΔNP63α, KRT12, KRT3, PAX6, and KRT14 in human cornea and organoids. Scar bar: 50 μm. (B) Immunofluorescent staining for ABCG2, Ki67, and MUC1 in human epithelial organoids. Scar bar: 50 μm. (C) The percentages of ΔNP63α^+^, ABCG2^+^, KRT12^+^, Ki67^+^cells in outer layer and inner cells, respectively. **p < 0.01. (D) Heatmap indicating the expression of corneal epithelial markers and certain functional genes in human epithelium tissue, organoids and HCECs. (E) Heatmap showing the expression niche cells markers in human epithelium tissue, organoids and HCECs.

In human corneal epithelium tissue, the consensual limbal stem cell marker ΔNP63α is mainly expressed in the basal layer, while KRT12^+^ keratinocytes are consistently located in the apical layer^16^ (Figure 2A). Similarly, the organoids preserved the characteristics of corneal epithelium well, with ΔNP63α^+^ cells lining the outer layer and KRT12 primarily expressed in the inner cells (Figure 2C). Other corneal epithelial markers, including KRT14, KRT3, ABCG2 and PAX6, were also found in the organoids (Figure 2A,B), showing an equivalent distribution to the tissue. Moreover, the proliferative marker Ki67 and the functional protein mucin1 (MUC1) were identified in the organoids (Figure 2A–C). Different distributions of markers indicated that the outer layer of the organoids aggregated into the stem cell‐like cells, while the inner cells were differentiated keratinocytes.

To further characterize the transcriptomic similarity between human tissue and organoids, we performed mRNA‐seq and used a 2D‐cultured human corneal epithelial cell (HCEC) line as a control. The heatmap showed that organoids and tissues shared similar expression levels of corneal limbal epithelial markers such as PAX6, TP63, KRT5, KRT14, KRT12, and KRT3, whereas those of HCECs were significantly different (Figure 2D). Interestingly, some biomarkers of the corneal niche cells, including VIM, MLANA, THY1 (CD90), KIT (CD117), ACTA2^17^ were also expressed in the organoids (Figure 2E), and their expression levels were closer to those in tissue than those in the 2D cell line.

**FIGURE 3:**
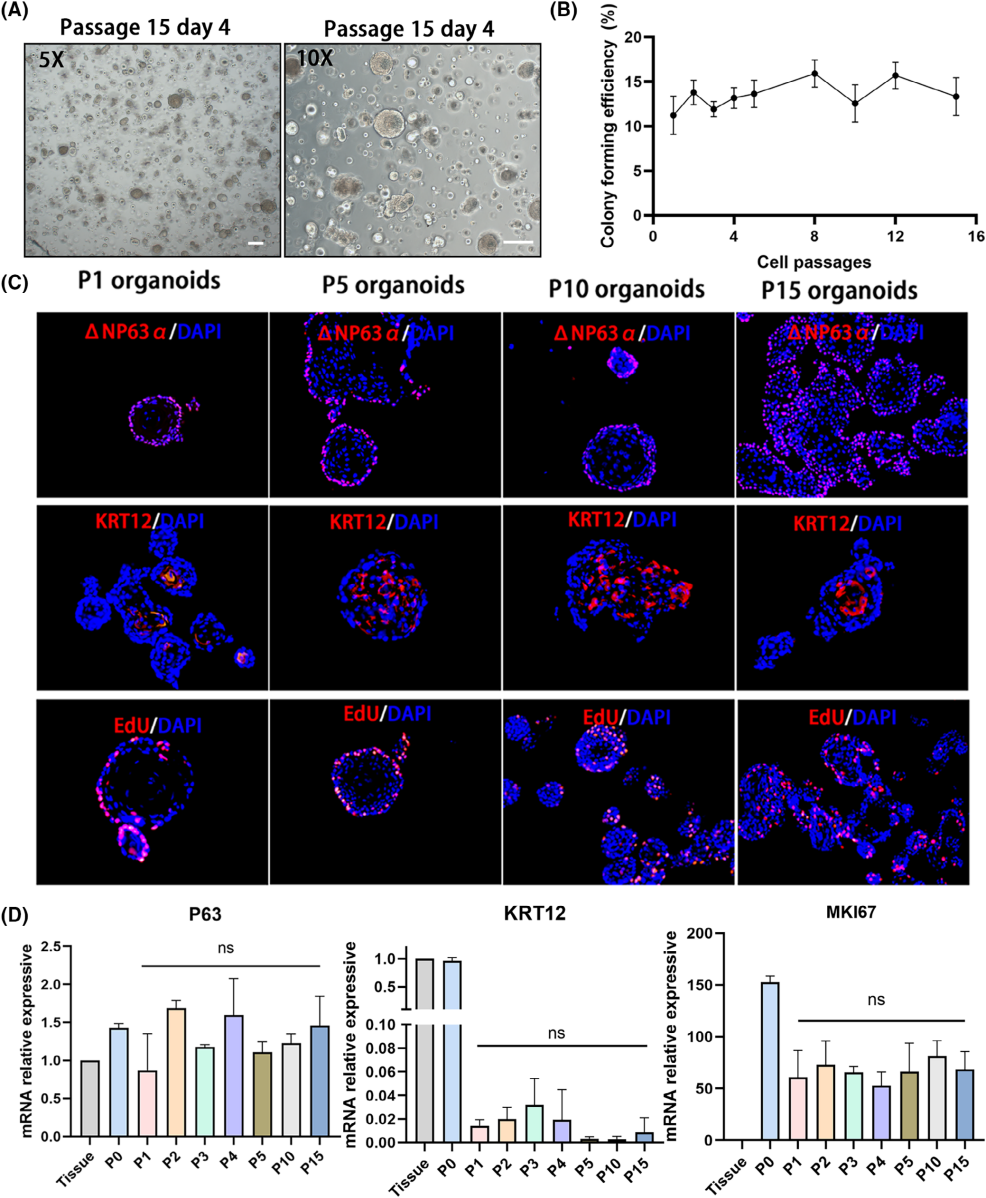
Long‐term culture of human epithelial organoids. (A) Representative images of organoids outgrowth in passage 15. Scar bar: 100 μm. (B) The colony forming efficiency of human organoids up to passage 15. (C) Immunofluorescent staining for ΔNP63α, KRT12, and EdU assay in P1, P5, P10 and P15 organoids. (D) qPCR analysis of the mRNA levels of P63, KRT12, and MKI67 in primary tissues and P0–P5, P10 and P15 organoids. ns: *p* > 0.05. P, passage.

Organoids were passaged at a 1:3 ratio every 6 days, and their morphology, features, and behaviour were maintained for more than 15 passages. (Figure 3A). During organoid passaging, the colony forming efficiency maintained stability (Figure 3B). IF staining and real‐time quantitative PCR (qPCR) revealed that the expression levels of ΔNP63α and KRT12 were stable during passaging. The percentage of proliferative (EdU^+^) cells and the mRNA levels of MKI67 showed no significant change in our organoid culture system (Figure 3C,D). Taken together, we established a long‐term functional human corneal epithelial organoid that recapitulated the cell lineages and gene expression signatures of the human corneal epithelium.

### 2.2 Differentiation of human corneal epithelial organoids

**FIGURE 4:**
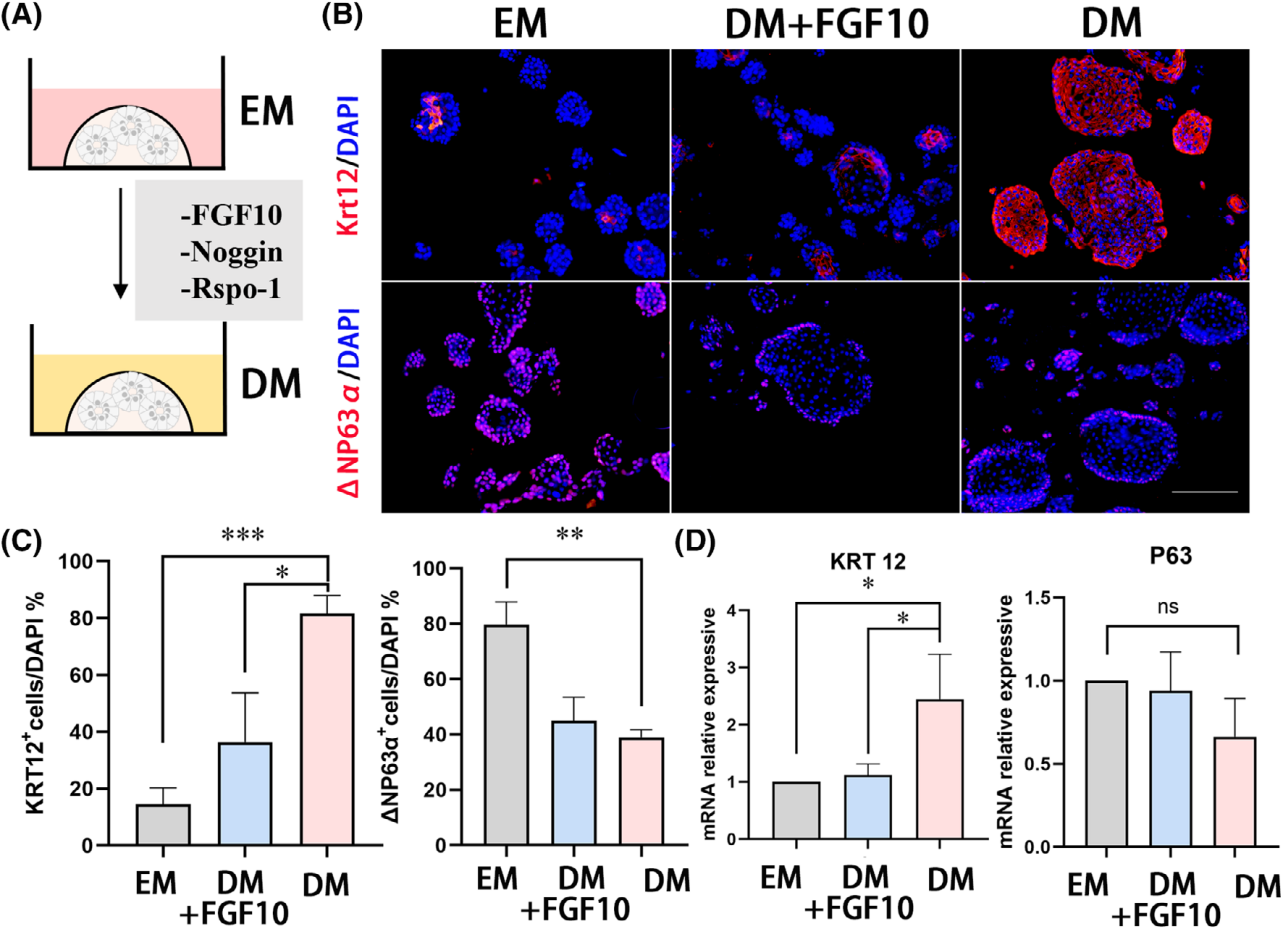
Regulation of human epithelial organoid differentiation. (A) Schematic of human epithelial organoid differentiation. (B) Immunofluorescent staining for ΔNP63α and KRT12 in expansion medium (EM), EM + FGF10 and differentiation medium (DM) treated groups. Scar bar: 50 μm. (C) The percentage of KRT12^+^ and ΔNP63α^+^ cells in EM, DM + FGF10 and DM groups. Data points represent biological replicates. Error bars indicate mean ± SD. *p < 0.05; **p < 0.01; ***p < 0.001. (D) qPCR analysis of the mRNA expression of KRT12 and P63 in EM, DM + FGF10 and DM groups. Data points represent biological replicates and error bars indicate mean ± SD. *p < 0.05.

During the culture of organoids, we observed that prolonging the culture period or removing the growth factors FGF10, Noggin, and Rspo‐1 for 3 days induced the differentiation of human corneal organoids (Figure 4A), resulting in a significant upregulation of KRT12^+^ cells (14.5%–81.7%, *p* < 0.001) and decreased expression of ΔNP63α (79.7%–39.1%, *p* = 0.001, Figure 4B,C). Although the mRNA level of P63 showed a slightly downward trend, the expression of KRT12 increased significantly (*p* = 0.032; Figure 4D). Thus, we used this system as a differentiation medium (DM).

To investigate the effect of FGF10 on the regulation of human epithelial organoids, we added FGF10 in DM. Significant downregulation of KRT12 in organoids was noted (from 81.7% to 36.4%, *p* = 0.013), and the expression of ΔNP63α increased from 39.1% to 45.0% (*p* = 0.310, Figure 4C). In summary, DM induced organoid differentiation, and FGF10 addition partly inhibited this function.

### 2.3 Human corneal epithelial organoids as a platform for ex vivo dry eye disease modelling

Dry eye is one of the most common ocular surface diseases of the corneal epithelium.^18^ The underlying causes of DED are complex and multifactorial. Thus, despite its high incidences and impact, ex vivo models of DED was limited, hindering studies on DED.^19^, ^20^ Herein, we developed a DED organoid model for disease exploration.

**FIGURE 5:**
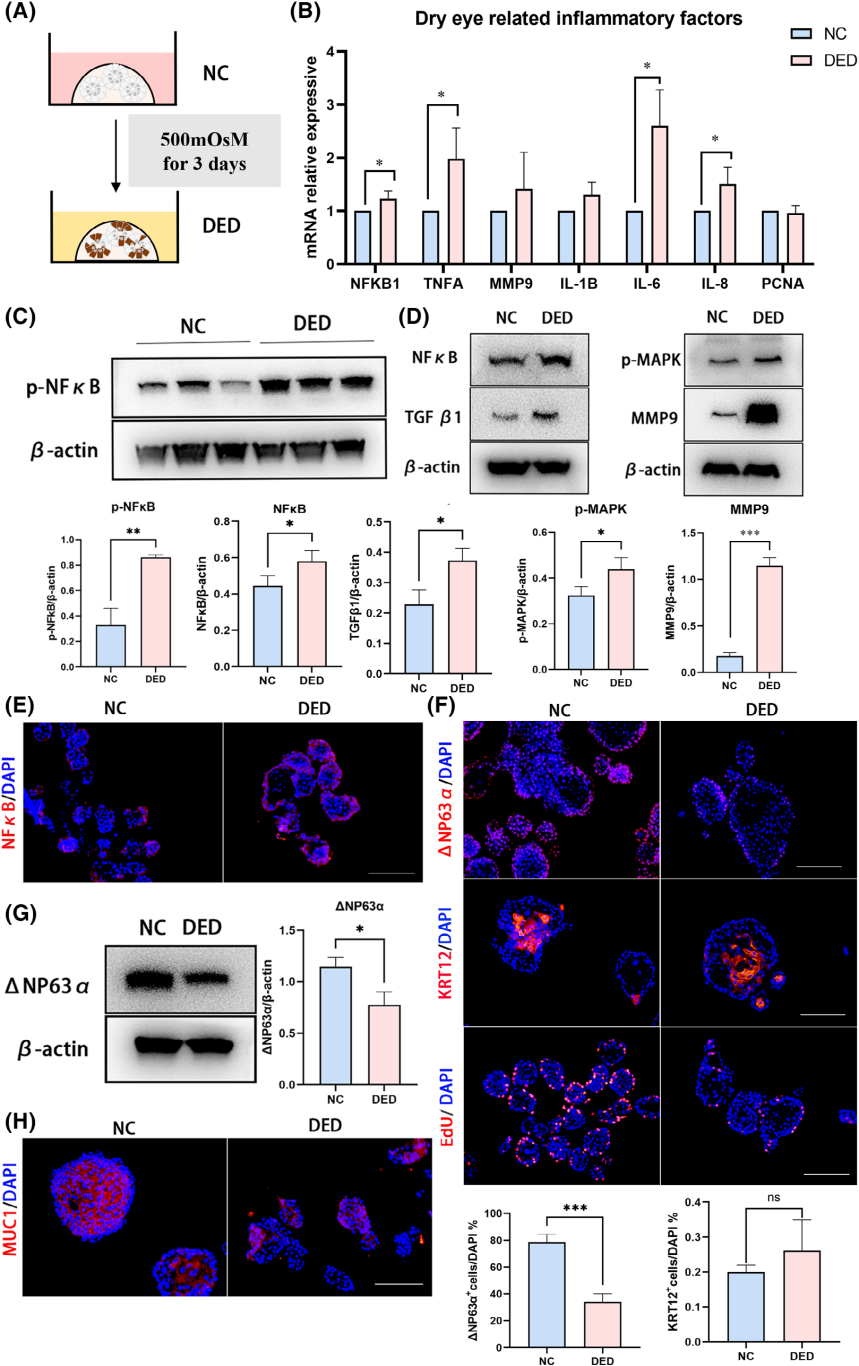
Hyperosmotic medium induced‐dry eye organoid model. (A) Schematic of dry eye disease (DED) organoid model induced by hyperosmotic medium. (B) The mRNA levels of dry eye related inflammatory factors including NFKB1, TNFA, MMP9, IL‐1B, IL‐6 and IL‐8, the expression of PCNA were also detected. *p < 0.05. (C) Western blot (WB) analysis and semiquantitative analysis the expression levels of p‐NFκB in normal control (NC) and DED groups. **p < 0.01. (D) WB analysis and semiquantitative analysis of the expression of NFκB, TGFβ, p‐MAPK and MMP9 in normal control (NC) and DED groups. *p < 0.05; ***p < 0.001. (E) Immunofluorescent staining for NFκB in NC and DED groups. Scar bar: 100 μm. (F) Immunofluorescent staining and quantitative analysis of ΔNP63α and KRT12 markers expression, and the EdU analysis in NC and DED groups. Scar bar: 100 μm. ***p < 0.001. (G) WB analysis and semiquantitative analysis of the protein expression of ΔNP63α in NC and DED groups. *p < 0.05. (H) Immunofluorescent staining for MUC1 expression in NC and DED groups. Scar bar: 100 μm.

Hyperosmotic stress was induced to establish a DED organoid model (Figure 5A). After 3 days of treatment, dry eye‐related inflammatory factors were detected with qPCR. In DED model, mRNA levels of NFKB1 (*p* = 0.043), TNFA (*p* = 0.042), IL‐6 (*p* = 0.016) and IL‐8 (*p* = 0.047) showed a significant increase (Figure 5B). Western blot (WB) analysis and IF staining revealed increased the expression of NFκB in DED model (Figure 5D,E), as well the significant upregulation of its activation form p‐NFκB (*p* = 0.002, Figure 5C). Other inflammatory factors in DED including TGFβ1 (*p* = 0.015) and p‐MAPK (*p* = 0.037) were increased, and the important biomarker matrix metalloproteinase 9 (MMP9, *p* < 0.001)^21^ upregulated significantly in DED organoid model.

In our organoid model, we found that the expression level of ΔNP63α decreased significantly (IF staining: *p* < 0.001; WB: *p* = 0.014), whereas the percentage of KRT12^+^ cells slightly increased in the DED model (Figure 5F,G). The decrease in EdU^+^ cells indicated that DED inhibits cell proliferation (Figure 5F). In addition, reduced mucin production protein (MUC1), suggests that our DED organoid model can reflect functional dry eye characteristics (Figure 5H). Therefore, we determined that human epithelial organoids could model DED and be used for drug development.

### 2.4 Human corneal epithelial organoids precisely mimicked the drug response of corneal epithelium

To evaluate the potential function of human corneal epithelial organoids in drug testing, we treated the human corneal organoids with three different drug: (1) Diquafosol ophthalmic solution (Diquas) is a P2Y2 receptor agonist that promotes tear and mucin secretion in dry eye patients^22^; Diquas treatment inhibits apoptosis and inflammation in the corneal epithelium in an animal model of DED.^23^ (2) Cyclosporine A (CsA), a calcineurin inhibitor, is an immunomodulatory drug that downregulates pro‐inflammatory cytokines and has therefore become the first‐line drug for DED.^24^ (3) Recombinant bovine basic fibroblast growth factor (bFGF) is safe and effective for the clinical treatment of DED promoting tear secretion and repairing corneal damage.^25^ Thus, three clinical drugs Diquas, CsA, and bFGF were added to the DED organoid model to mimic the drug response in the human corneal epithelium.

**FIGURE 6:**
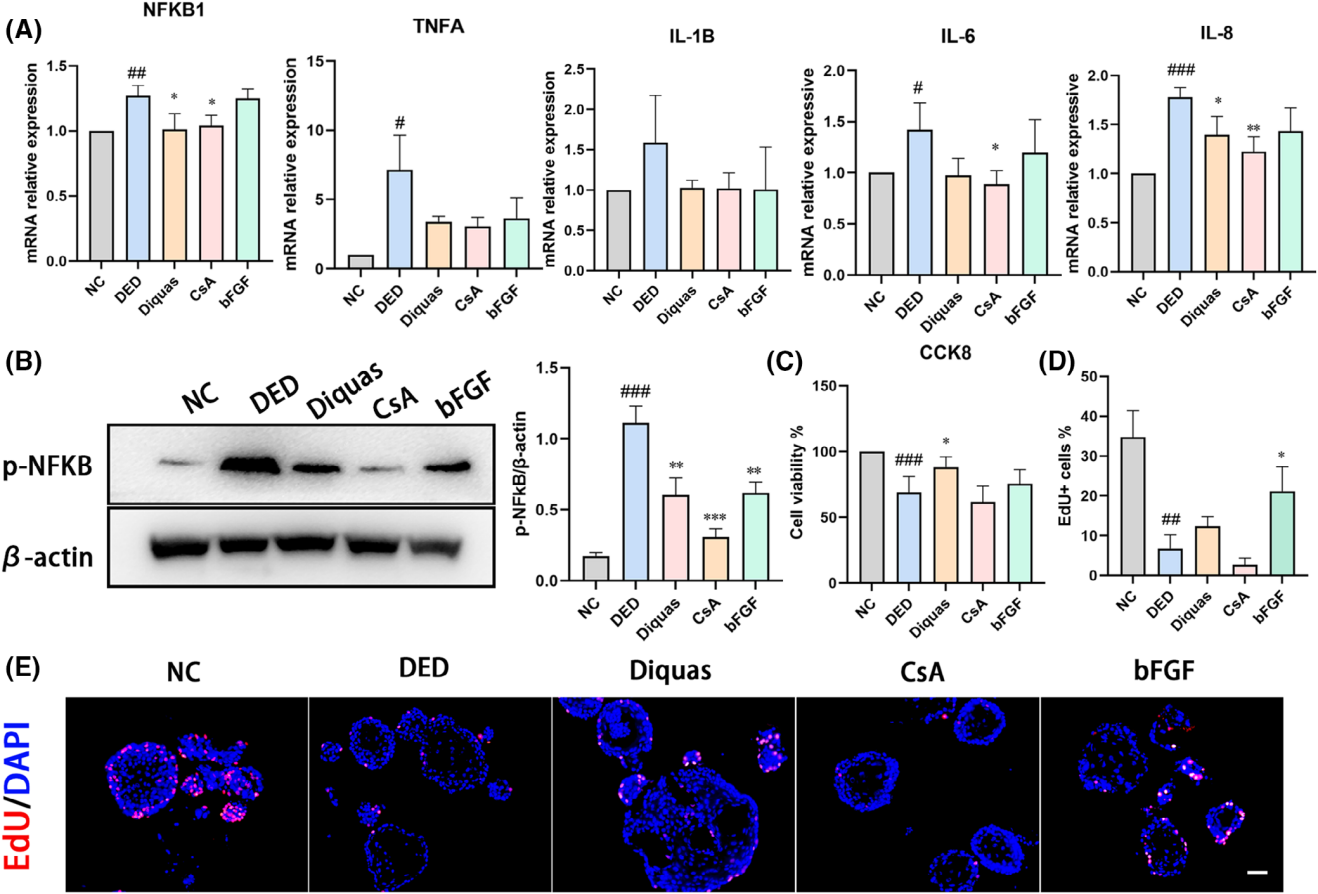
Drug testing in DED organoids. (A) The mRNA expression levels of NFKB1, TNFA, IL‐1B, IL‐6 and IL‐8 in the indicated treated groups. NC: normal control, DED: dry eye disease, Diquas: 0.3% Diquafosol tetrasodium, CsA: 10 nM CsA, bFGF:100 ng/mL bFGF. # showed DED compared with NC group, * was the drug treated group compared with DED. #*p* < 0.05; ##*p* < 0.01; ###*p* < 0.001; *p < 0.05; **p < 0.01. (B) WB analysis and semiquantitative analysis of the expression of p‐NFκB in the indicated groups. # showed the DED compared with NC group, * was the drug treated group compared with DED. ###*p* < 0.001; **p < 0.01; ***p < 0.001. (C) CCK‐8 analysis of cell viability in the indicated groups. # showed the DED compared with NC group, * was the drug treated group compared with DED. ###*p* < 0.001; *p < 0.05. (D) The percentage of EdU^+^ cells in different drug treated groups. # showed the DED compared with NC group, ##*p* < 0.01. (E) Representative images of EdU assay in different drug therapy groups. Scar bar: 50 μm.

After treatment for 3 days, CsA displayed evident anti‐inflammation effects, which significantly inhibited the expression of NFKB1 (*p* = 0.024), IL‐6 (*p* = 0.035) and IL‐8 (*p* = 0.006). In WB analysis, the expression of p‐NFκB downregulated to the level of normal control (Figure 6A,B). However, CsA treatment slightly decreased cell viability and proliferation compared to the DED group (Figure 6C–E). Diquas reduced the mRNA levels of NFKB1 (*p* = 0.037) and IL‐8 (*p* = 0.034), downregulated p‐NFκB in WB analysis (Figure 6A,B), and demonstrated the ability to enhance the cell viability (*p* = 0.017) and increase EdU^+^ cells (Figure 6C–E). The addition of bFGF slightly inhibited the expression of pro‐inflammatory cytokines while significantly promoting cell proliferation in DED model (EdU^+^ cells increased from 6.8% to 21.2%, *p* = 0.023). In summary, our results suggest that epithelial organoids can mimic drug responses in animal models and human clinical trial reports.^22^, ^23^, ^24^, ^25^, ^26^

## 3 DISCUSSION

For decades, several efforts have been made to develop a suitable corneal epithelial culture model. Organoid cultures have the potential to explore organ‐level biology ex vivo and can mimic physiology and pathology more closely than 2D cell culture systems.^27^ With regard to the ocular surface, ASCs‐derived organoids are developed rapidly. In 2021, the first human lacrimal gland organoids were established and could phenocopy the process of tear secretion ex vivo.^13^ Subsequently, human conjunctival organoids have been generated, providing a platform for studying ocular surface homeostasis and diseases.^9^ Furthermore, in our primary study, we successfully generated the human meibomian gland organoids (under review), suggesting the exploration of epithelial homeostasis and dysfunction of meibomian glands and facilitating drug development and regenerative medicine for dry eye disease.

In the current study, we developed a long‐term ASC‐derived 3D corneal epithelial organoids culture system. The morphology, transcription, and function of the organoids recapitulated the characteristics of the human corneal epithelium. Moreover, our expansion culture system maintained the stable characteristics of organoids for a long‐term (>3 months), and switching to the differentiation medium promoted the organoid differentiation, suggesting that organoids could serve as useful tools to explore the homeostasis of the corneal epithelium. Matrigel, a basement membrane matrix extracted from extracellular matrix protein‐rich EHS mouse tours containing approximately 60% laminin, 30% IV collagen and 8% nestin, which has the advantages of versatility and affordability, commercially available, extensively used with well‐developed protocols,^28^ was used for the supporting of organoid culture in this study. The WNT, BMP and FGF signalling pathways may play important roles in corneal epithelial proliferation and differentiation. To inhibit the differentiation of organoids, it is crucial to focus on the functional regulation of the corneal epithelium through the FGF10 signalling pathway. FGF10 has been confirmed to be involved in eye, meibomian gland and lacrimal gland development, and is able to increase the viability and proliferation of corneal epithelial cells.^29^ The mechanism underlying the FGF10 signalling pathway in the corneal epithelium is of interest.

DED is a common ocular surface disease that has become a crucial healthcare concern worldwide and affects the global socio‐economic development.^3^ In vitro experiments enable precise control over microphysio‐pathological conditions, making them invaluable for preclinical and thereby reducing the requirement of animal studies.^30^ Traditionally, DED‐like symptoms are induced by the treatment of hyperosmotic media and supplementation of corneal epithelial cells with pro‐inflammatory cytokine in vitro.^31^, ^32^ Several DED models based on new system or technology were developed ex vivo or in vitro recently. For example, Meloni et al. exposed the reconstruct 3D corneal epithelium to dryness, and co‐incubated them with human leukaemia monocytic cell line to develop an in vitro model of severe immunocompetent‐DED.^33^ Also, transferring pro‐inflammatory factor‐treated HCEC to a microfluidic device can establish a dynamic inflammatory DED model.^34^ Using an organ‐on‐chip device growing HCEC under the application of cycles of liquid stimuli can be performed to mimic the tear‐like evaporation process.^20^ Although DED in vitro models are emerging recently, tear film osmolarity is still considered as the core factor in the pathogenesis of dry eye.^18^ Tear hyperosmolarity triggers a cascade of inflammatory events, leading to the activation of JNK1/2, MAPK, NFκB signalling pathway, and the releases of pro‐inflammatory factors, resulting from decreased lacrimal flow or tear film breakup contribute to ocular surface damage.^18^, ^35^ Therefore, in this study, we performed the hyperosmotic stress to explore the feasibility of corneal epithelial organoid for DED modelling. Here we found not only do dry eye‐related inflammatory factors such as NFκB, IL‐6 and IL‐8 show significant activation,^18^, ^36^ but the proliferation and stemness of limbal stem cells, along with the visualization of functional mucin protein, can also be observed in this model. This offers the potential to explore the stem cell identity and keratinocyte function in DED. Lin et al. addressed that severe dry eye could induce abnormal differentiation and loss of stemness in the corneal epithelium of an animal model,^37^ which is consistent with our findings. Damage to limbal stem cells can aggravate DED. A clinical trial found the treatment with corneal epithelial stem cell supernatant in patients with severe dry eye can improve their symptoms and quality of life,^38^ suggesting the corneal epithelial organoids are promising alternatives for unresponsive patients with severe DED.

Similar to that observed in previous study, CsA and Diquas displayed significant anti‐inflammatory effects, and bFGF10 promoted the cell proliferation in DED organoid model.^26^, ^39^ These results suggests that the organoids have the potential to establish a drug‐screening platform to evaluate the effectiveness and safety of stem cells, niche cells and keratinocytes. In addition to drug development for DED, patients' derived organoids promote a clearer biological understanding of individualized treatment options for ocular surface disease. In addition, as described by Higa et al., corneal limbal organoids show promise for epithelial cell transplantation in limbal stem cell deficient animals, highlighting the potential of organoids to pave the way for regenerative medicine in ocular surface disease.^8^, ^12^ Gene editing is another promising application of corneal epithelial organoids. Targeting the TGFBI gene in organoids derived from patients with corneal dystrophy may solve the challenges of phenotypic instability in animal models, and provide a useful tool for evaluating gene editing ex vivo.

This study had certain limitations. First, ASCs show limited differentiation potential, therefore the ASCs‐based organoids only represented epithelial structure in our study. Corneal organoids derived from induced pluripotent stem cells express the key epithelial, stromal and endothelial cell markers.^11^ However, with the advantages of fewer step to culture, more consistent reproduction of the original tissue phenotype and have the ability to establish malignant tumour organoids of ASCs‐based organoids,^40^ combining the exploration of induced pluripotent stem cells with ASCs‐derived organoids could significantly advance studies of the ocular surface. In the present study, we developed a hyperosmolar‐induced organoid DED model. Whether epithelial organoids can be treated with other stimuli to closely mimic the feature of DED should be explored in further studies.

In conclusion, we established functional, long‐term cultured 3D human corneal epithelial organoids, which could serve as a useful tool for the ex vivo exploration of corneal epithelial homeostasis and disease.

## 4 METHODS

### 4.1 Human samples

Cadaveric human corneal tissues aged 30–70 years, were obtained from the eye bank of the Eye, Ear, Nose and Throat Hospital of Fudan University. All the subjects provided written informed consent to participate in this study. All the tissue used for organoids culture did not meet the criteria for clinical use and the research was conducted with the Declaration of Helsinki and approved by the Ethics Committee of the EENT Hospital of Fudan University (EENT‐IRB2022‐02‐22).

### 4.2 Human corneal epithelial organoid cultures

Human corneal epithelial tissue including limbal epithelium was used for corneal epithelial organoids culture. After washing in phosphate‐buffered saline (PBS), the excess sclera, conjunctiva, iris, trabecular meshwork and corneal endothelium were carefully removed. The corneal tissues were then placed on culture plates with the epithelium facing down, and 1 mg/mL collagenase I with 10 μM Y‐27632 and 20 U/mL DNase I were used for digestion. Following 1 h of digestion, epithelial cells were scraped off carefully. The digestion solution was washed twice with Advanced DMEM/F12 (Gibco, USA), and Growth Factor Reduced Matrigel (Corning, USA) was added to resuspend the cells on ice. Then, the mixture was dropped on the plate, and incubated in 37°C for 10 min. After the Matrigel solidification, growth medium was performed for organoid culture.

Advanced DMEM/F12 (Gibco, USA) supplemented with 1xB27 (Gibco, USA), Glutamax (Gibco, USA), HEPES (Gibco, USA), 100 U/mL Penicillin Streptomycin (Gibco, USA), 1.25 mM N‐acetylcysteine (Sigma‐Aldrich, USA), 10 mM nicotinamide (Sigma‐Aldrich, USA), 0.5 μM A83‐01 (OrganRegen), 1 μM PGE2 (OrganRegen), 1 μM FSK (OrganRegen), 100 ng/mL FGF10 (OrganRegen), 100 ng/mL Noggin (OrganRegen), and 100 ng/mL Rspo‐1 (OrganRegen) contained the human expansion medium. The removal of growth factors 100 ng/mL FGF10 (OrganRegen), 100 ng/mL noggin (OrganRegen), and 100 ng/mL Rspo‐1 (OrganRegen) from the expansion medium constitutes the differentiation medium. After 6 days of expansion culture, the organoids were mechanically dissociated into small fragments, and TrypLE Express (Gibco, USA) with 1 mM EDTA (Gibco, USA) were used for digestion. The organoids were passaged at a ratio of 1:2–3, and the dissociated human epithelial cells were embedded into fresh Matrigel. The organoids were incubated at 37°C with 5% CO_2_, and the medium was changed every 2–3 days.

### 4.3 IF staining

The organoids were harvested after 6 days of culture, fixed with 4% paraformaldehyde (Sigma‐Aldrich, USA) for 30 min at room temperature, and dehydrated in 30% sucrose for 1 h at 4°C. For human cornea, the tissues were fixed and dehydrated overnight at 4°C. Then, the samples were embedded in optimum cutting temperature compound (OCT, SAKURA), and the embedded sample were cut into 10‐μm‐thick sections before IF staining. Both organoids and tissues were treated with 0.3% Triton X‐100 for 10 min to permeabilize followed by blocking with 3% BSA for 30 min at room temperature. Subsequently, the samples were incubated with primary antibodies overnight at 4°C. After three washes in PBS, the samples were incubated with secondary antibodies at room temperature for 1 h, and the nuclei were labelled using Hoechst 33258 (Invitrogen, 1:2000). The images were acquired with fluorescence microscopy (Zeiss, Scope. A1, Germany), and quantification of staining positive cells were performed using Image J software (NIH, Washington, DC).

### 4.4 Colony formation assay

After dissociating the organoids into single cells, 2000 cells were seeded in a 96‐well plate and cultured for 6 days. The number of organoids was counted, and the colony‐forming efficiency was calculated using the following formula: (number of organoids)/2000 × 100%.

### 4.5 Dry eye organoids model and drug testing

Hyperosmolar exposure was used to establish the DED organoid model. Organoids initially cultured in isosmotic conditions (312mOsM) were transferred to hyperosmotic culture medium (500mOsM) for 3 days. Hyperosmolar stress was induced by treatment with 94 mM sodium chloride.

For drug testing, 0.3% diquafosol tetrasodium (MedChemExpress, USA), 10 nM CsA (MedChemExpress, USA) and 100 ng/mL FGF basic/bFGF Protein (MedChemExpress, USA) were performed to identified the organoids' response to different drugs. After 3‐day co‐incubation with DED organoids, the organoids were harvested for further experiments.

### 4.6 qPCR analysis

The organoids were collected after 6 days of cultures, and the total RNA were extracted using the Universal RNA Purification Kit (EZBioscience, USA) according to the manufacturer's protocol. The Colour Reverse Transcription Kit (EZBioscience, USA) were used for reverse transcription of cDNA. Then, qRT‐PCR were performed with 2* Colour SYBR Green qPCR Master Mix kit (EZBioscience, USA). The target gene mRNA expression levels were normalized to β‐actin, and evaluated by the 2^−ΔΔCt^ method. The sequences of the target gene‐specific primers are shown in Table S1.

### 4.7 WB analysis

After 6 days of culturing, the organoids were harvested. RIPA buffer (Beyotime Biotechnology) was used to lyse cells and obtain the total protein. BCA Protein Assay kit (Beyotime Biotechnology, China) was performed to detect the protein concentration according to the manufacturer's protocol. 4%–20% SDS‐polyacrylamide gel electrophoresis (GenScript, China) was used to separate the protein, and the gel was transferred to a PVDF membrane (Millipore, USA). After being blocked with 5% non‐fat powdered milk, the membrane was incubated with primary antibodies overnight at 4°C. After a thorough wash with Tris‐buffered saline with Tween (TBST), HRP‐conjugated secondary antibodies were incubated with the membranes at room temperature for 1 h. β‐actin were used as internal reference for total protein. Immobilon Western HRP Substrate (Millipore, USA) was used for chemiluminescent detection, and the protein expression levels were semi‐quantified with Image J software (NIH, Washington, DC). The primary and secondary antibodies are shown in Table S2.

### 4.8 EdU assay

An EdU cell proliferation kit (Beyotime Biotechnology, China) was used to detect proliferating cells in organoids. The 10 μM EdU solution were added into 6‐day organoids followed by coincubation for 4 h. After that, the organoids in 24 wells were fixed with 4% paraformaldehyde, dehydrated and embedded. The OCT embedded organoids were cut into 10‐μm‐thick sections for staining. Then, the click relative solution was added to cross‐link EdU with the fluorescent azide, which can be detected under a fluorescence microscope. The cell nuclei were labelled using Hoechst 33258 (Invitrogen, 1:2000), and the percentage of EdU positive cells was calculated to assess the cell proliferation of organoids.

### 4.9 Cell viability assay

According to the manufacturer's protocol, a Cell Counting Kit‐8 (CCK‐8) assay kit (Dojindo, Kumamoto, Japan) was used to detect the effect of drugs on the cell viability of organoids. After 3‐day treatment, 100 μL organoid cultured medium with CCK‐8 solution was co‐incubated for 2 h. The absorption was read at 450 nm using the microplate reader (TECAN spark) and the cell viability was calculated by the following formula: Cell viability (%) = [(As − Ab)/(Ac − Ab)] × 100%; As, Ac and Ab are the absorbance of experimental, control and blank group, respectively.

### 4.10 mRNA‐sequencing

Human corneal epithelial cell line (HCEC), corneal epithelial organoids and corneal epithelium tissue were performed for mRNA‐sequencing analysis. For tissue, human corneas were collected and incubated with 2.4 U/mL Dispase II (Roche) at 37°C for 2 h. Then, corneal epithelium was peeled away carefully. The total RNA of tissue, organoids and HCEC were extracted as described above.

A total of 1 μg high‐quality RNA of each sample was performed to construct sequencing libraries using a TruSeq RNA Sample Preparation Kit (Illumina), and more than 40 million reading were obtained for each sample. After quantification using a TBS380 fluorometer, the RNA sequencing library was sequenced used an illumina HiSeq X Ten/NovaSeq sequencer. Differential gene expression was evaluated using edgeR. Genes with |log2FC| > 1 and a *Q* value %0.05 were considered to be significantly differentially expressed.

### 4.11 Statistical analysis

Three replicates were performed and the results data are shown as the mean ± SD. GraphPad Prism software (La Jolla, CA, Version 9) was used for statistical analysis. Student's *t* test was used for comparisons between two groups. The *p* values less than 0.05 were considered statistically significant.

## AUTHOR CONTRIBUTIONS

X.W., Y.H. and J.H. conceived the study; X.W., J.G., Q.L., X.Z., W.J. and X.C. performed the experiments; Y.H. and J.H. supervised the work; and X.W., Y.H. and J.H. wrote the manuscript.

## CONFLICT OF INTEREST STATEMENT

The authors declare no conflicts of interest.

## Supporting information


**Figure S1.**The schematic diagram of the research.

**Data S1. Table S1.** The specific primer sequences.
**Table S2.** The antibodies and manufacturer.

## Data Availability

The data that support the findings of this study are openly available in Sequence Read Archive (SRA) at https://submit.ncbi.nlm.nih.gov/subs/sra/, reference number PRJNA1093384.
