# Supplementary material for: Establishment of human corneal epithelial organoids for ex vivo modelling dry eye disease
Source: Cell Prolif. 2024 Jul 3;57(11):e13704. doi: 10.1111/cpr.13704 (PMC11533071; doi:10.1111/cpr.13704)
Supplement: Supplementary file 1 — Figure S1.The schematic diagram of the research. [file CPR-57-e13704-s001.docx]

**
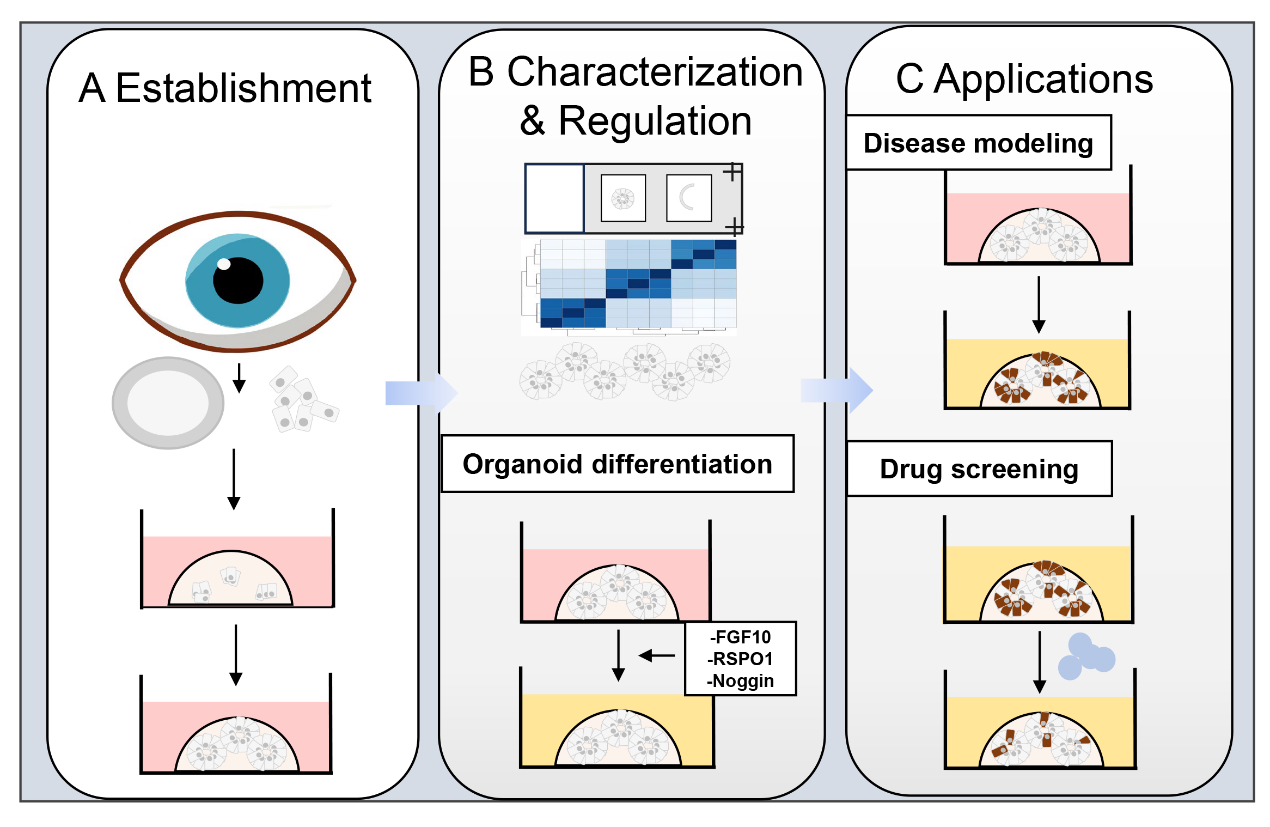
Supplementary figure 1**

**Figure legend**

**The schematic diagram of the research**

The culture system of human epithelial organoids was generated (A). Then, the similarity between tissue and organoids were characterized, and the regulation of organoid differentiation were explored (B). Finally, the DED organoid model was established and evaluated, and the DED drug testing platform was built *ex vivo* (C).
