# Supplementary material for: Establishment of human corneal epithelial organoids for ex vivo modelling dry eye disease
Source: Cell Prolif. 2024 Jul 3;57(11):e13704. doi: 10.1111/cpr.13704 (PMC11533071; doi:10.1111/cpr.13704)
Supplement: Supplementary file 2 — Data S1. Table S1. The specific primer sequences. Table S2. The antibodies and manufacturer. [file CPR-57-e13704-s002.docx]

**Table S1: The specific primer sequences**

| **Gene** | **Primer** | **Sequences (5’-3’)** |
| --- | --- | --- |
| **Human** |  |  |
| TP63 | Forward | GGACCAGCAGATTCAGAACGG |
| TP63 | Reverse | AGGACACGTCGAAACTGTGC |
| KRT12 | Forward | CTCTCCTCGCAGAGTGTGATA |
| KRT12 | Reverse | AACTAGAACCAAACATGGAAGCA |
| MKI67 | Forward | AGAAGAAGTGGTGCTTCGGAA |
| MKI67 | Reverse | AGTTTGCGTGGCCTGTACTAA |
| NKFB1 | Forward | AACAGAGAGGATTTCGTTTCCG |
| NKFB1 | Reverse | TTTGACCTGAGGGTAAGACTTCT |
| PCNA | Forward | CCTGCTGGGATATTAGCTCCA |
| PCNA | Reverse | CAGCGGTAGGTGTCGAAGC |
| TNF | Forward | CCTCTCTCTAATCAGCCCTCTG |
| TNF | Reverse | GAGGACCTGGGAGTAGATGAG |
| MMP9 | Forward | TGTACCGCTATGGTTACACTCG |
| MMP9 | Reverse | GGCAGGGACAGTTGCTTCT |
| IL-1b | Forward | ATGATGGCTTATTACAGTGGCAA |
| IL-1b | Reverse | GTCGGAGATTCGTAGCTGGA |
| IL-6 | Forward | ACTCACCTCTTCAGAACGAATTG |
| IL-6 | Reverse | CCATCTTTGGAAGGTTCAGGTTG |
| IL-8 | Forward | ACTGAGAGTGATTGAGAGTGGAC |
| IL-8 | Reverse | AACCCTCTGCACCCAGTTTTC |
| ACTB | Forward | GTGGCCGAGGACTTTGATTG |
| ACTB | Reverse | CCTGTAACAACGCATCTCATATT |

**Table S2: the antibodies and manufacturer**

| **Antibody** | **Manufacturer** | **Identifier** |
| --- | --- | --- |
| Anti-β-actin | Sigma-Aldrich | A3854 |
| Anti- Cytokeratin 14 | Abcam | ab7800 |
| Anti-Ki-67 | BD Pharmingen | 550609 M |
| Anti- keratin 12 | Abcam | ab185627 |
| Anti- keratin 3 | Proteintech | A10118 |
| Anti- DeltaN P63 | CST | 67825 |
| Anti-PAX6 | Proteintech | A24118 |
| Anti-MUC1 | Abcam | ab109185 |
| Anti-ABCG2 | Abcam | ab207732 |
| Anti-NF-κB p65 | Proteintech | 10745-1-AP |
| Anti-phospho-NF-κB p65 | CST | 3033 |
| Anti-MMP9 | Abcam | 76003 |
| Anti-phospho-p38 MAPK | Proteintech | 28796-1-AP |
| Alexa Fluor 488 donkey anti-mouse | Thermo Fisher scientific | A21202 |
| Alexa Fluor 555 donkey anti-rabbit | Thermo Fisher scientific | A31572 |
| HRP-labeled Goat Anti-Rabbit IgG(H+L) | Beyotime | A0208 |
| HRP-labeled Goat Anti-Mouse IgG(H+L) | Beyotime | A0216 |
